# Panmixia in a Widespread Butterfly: High Dispersal and Ecological Generalism Buffer Against Landscape Fragmentation

**DOI:** 10.1002/ece3.74318

**Published:** 2026-09-03

**Authors:** Praveen Dhyani, Franziska Deppe, Klaus Fischer

**Affiliations:** ^1^ Department of Biology, Institute for Integrated Natural Sciences University of Koblenz Koblenz Germany

**Keywords:** agricultural landscapes, connectivity, gene flow, landscape genetics, *Pieris rapae*, SNPs

## Abstract

Habitat fragmentation is widely expected to reduce population connectivity and increase genetic differentiation, although the strength of these effects depends on species‐specific traits such as dispersal ability. Here, we investigated the population genetic structure of the cosmopolitan butterfly, *Pieris rapae* L. (Lepidoptera: Pieridae), across western Germany using genome‐wide single‐nucleotide polymorphism (SNP) data. To analyze the effects of landscape structure on genetic connectivity, we applied a paired study design comprising four landscape pairs, each consisting of a highly intensified, modern agricultural landscape and a more heterogeneous, traditional landscape. Our results revealed no evidence of genetic differentiation. Pairwise F_ST_ values were close to zero; we detected no isolation by distance, and clustering analyses supported a single genetic population. No meaningful associations between genetic variation and environmental variables were detected, with landscape effects explaining less than 0.4% of genomic variation. Consequently, we found no evidence for stronger genetic structuring in modern compared to more connected traditional landscapes. Our results suggest that extensive habitat fragmentation does not necessarily translate into reduced genetic connectivity in highly mobile, generalist species. In *P. rapae* , high dispersal ability and ecological generalism appear to buffer against the genetic consequences of landscape modification, resulting in panmictic population structure even across strongly contrasting agricultural landscapes.

## Graphical abstract

The study examined the genetic structure of *Pieris rapae* across western Germany and found no genetic differentiation or isolation by distance. Fragmented agricultural landscapes did not reduce genetic connectivity compared to more heterogeneous systems, indicating near‐panmixia across the region. The results highlight that highly mobile generalist species are little affected by habitat fragmentation.

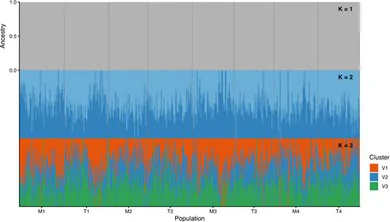

## 1 Introduction

Human activities have transformed ecosystems worldwide, resulting in a dramatic decline in global biodiversity (Ceballos et al. 2015, 2020). Land‐use change and the associated habitat loss and fragmentation are key drivers of ongoing biodiversity loss (Newbold et al. 2015; Montràs‐Janer et al. 2024). Consequently, natural habitats are increasingly converted into urban or intensively managed agricultural landscapes (Foley et al. 2005; Haddad et al. 2015). These processes reduce habitat area, increase isolation between habitat patches, and alter dispersal dynamics, thereby reshaping population connectivity and community composition (Foley et al. 2005; Newbold et al. 2015).

A widely observed consequence of these changes is biotic homogenization, whereby ecological communities become increasingly similar across space as generalist species spread and specialist taxa decline (McKinney and Lockwood 1999; Olden and Poff 2003). This pattern is closely linked to anthropogenic environmental filtering, which favors species with broad ecological niches, high disturbance tolerance, and strong dispersal ability (Devictor et al. 2008; Clavel et al. 2011). As a result, regional biotas lose functional and taxonomic distinctiveness, leading to reduced beta diversity across landscapes (Olden and Poff 2003; Newbold et al. 2015).

The effects of habitat fragmentation and land‐use change though are highly context‐ and trait‐dependent (Ewers and Didham 2006; Tscharntke et al. 2012). Species differ in their sensitivity to landscape modification, owing to variation in dispersal ability, habitat specialization, and reproductive strategy. In particular dispersal ability plays a central role in determining population connectivity, gene flow, and ultimately genetic structure across space (Bohonak 1999; Lenormand 2002). While habitat fragmentation often reduces gene flow and increases genetic differentiation in low‐dispersal species, highly mobile organisms may maintain connectivity even across strongly fragmented landscapes, thereby buffering against genetic structuring (Bohonak 1999; Lowe and Allendorf 2010; Storfer et al. 2010).

Butterflies are a model group for studying these processes because they exhibit pronounced interspecific variation in dispersal ability and, consequently, responses to land‐use change (Hanski 1999; Öckinger et al. 2010). Several studies have shown that butterfly diversity and community composition respond strongly to habitat loss and fragmentation, particularly in agricultural landscapes (Hanski 1999; Steffan‐Dewenter and Tscharntke 2000; Krauss et al. 2003). However, mobile generalists often persist in agricultural settings, while specialists typically decline (Dennis et al. 2003; Öckinger et al. 2010).

**FIGURE 1:**
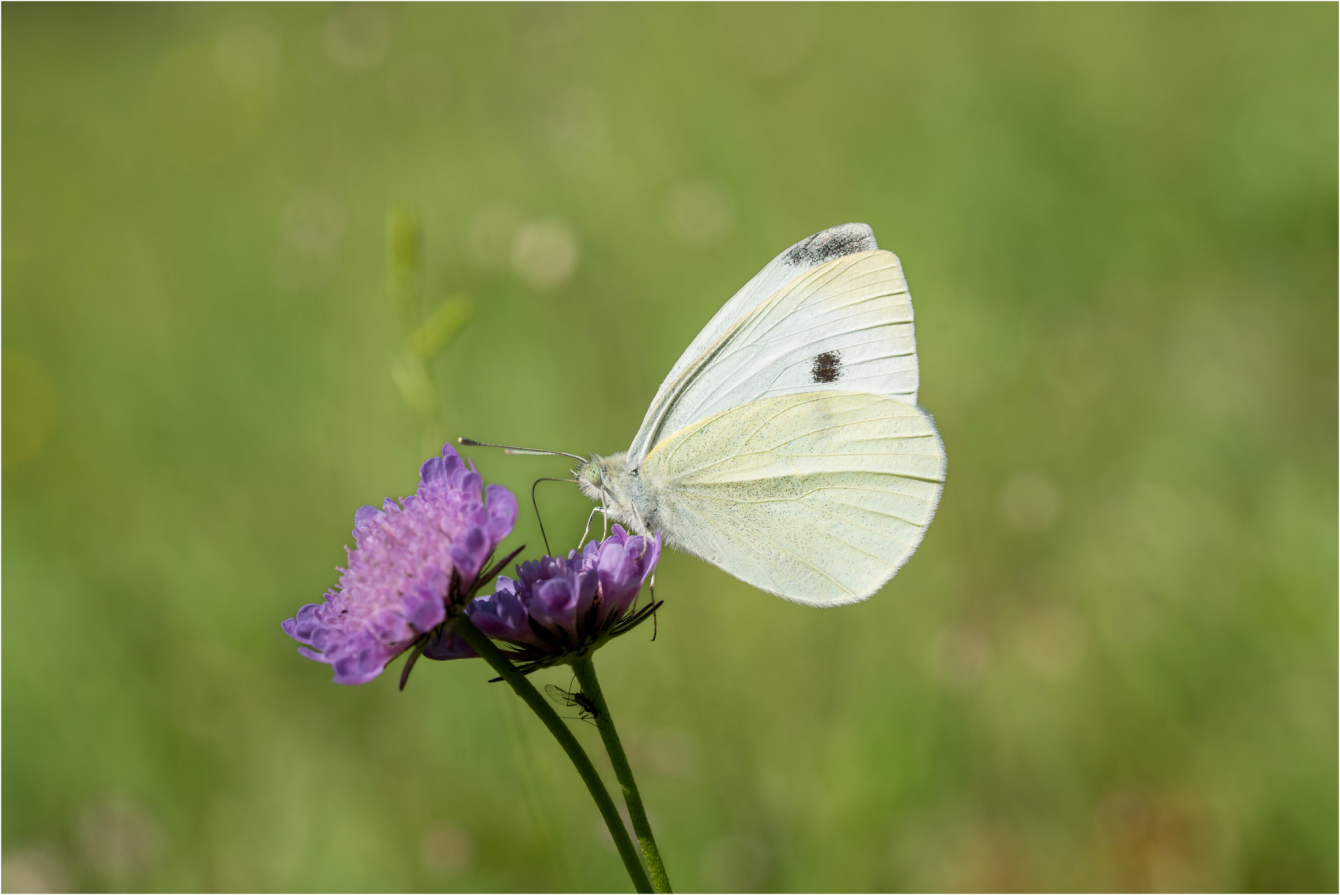
The study organism *Pieris rapae* (Lepidoptera: Pieridae). Photograph: Stella Buchwald.

Among butterflies, the Cabbage white, *P. rapae* , represents a cosmopolitan species which is one of the most common and widespread butterflies in Germany (Ryan et al. 2019; Figure 1). It is a highly successful example of an anthropogenically associated species (Ryan et al. 2019). It commonly occurs in agricultural landscapes, as it is able to use even eutrophic habitats (Chew and Renwick 1995; Maes and Van Dyck 2001). Larvae feed on a wide variety of Brassicaceae, including crops such as rapeseed, cabbage, or turnips (Ryan et al. 2019). The species is highly mobile, typically searching for suitable host plants within a radius of several kilometers (Ikeura et al. 2010). *P. rapae* typically has 3–4 generations per year, and it overwinters as pupae (Ryan et al. 2019). The species' high dispersal capacity, ecological generalism, and close association with human‐modified environments make it a suitable model system for examining whether extensive habitat fragmentation translates into detectable population genetic structure (Hill et al. 1999; Lowe and Allendorf 2010). Despite strong landscape modification across Europe, highly mobile species such as *P. rapae* may maintain near‐panmictic population structure due to effective long‐distance dispersal (Bohonak 1999; Lowe and Allendorf 2010).

In this study, we investigate the population genetic structure of *P. rapae* across western Germany using genome‐wide SNP data. To test whether landscape structure influences genetic connectivity, we employed a paired study design comprising four landscape pairs, each consisting of a highly intensified, structurally simplified ‘modern’ agricultural landscape and a more ‘traditional’, more complex landscape with smaller field sizes and higher habitat heterogeneity. Within each landscape, 10 sampling sites were analyzed to assess fine‐scale genetic structure. Given the species' high mobility and ecological generalism, we predict low genetic differentiation among populations (Bohonak 1999; Lowe and Allendorf 2010). However, if habitat fragmentation constrains dispersal even in this species, we expect stronger genetic structuring among sampling sites in modern, fragmented landscapes compared to more connected traditional landscapes (Manel et al. 2003; Ewers and Didham 2006; Storfer et al. 2010). More broadly, our study contributes to understanding how species traits mediate genetic responses to anthropogenic landscape change (Tscharntke et al. 2012; Clobert et al. 2013).

## 2 Materials and Methods

### 2.1 Study Organism

We used the Cabbage white *P. rapae* to investigate the effects of landscape structure and fragmentation on population genetic structure.

### 2.2 Study Area and Sample Collection

**FIGURE 2:**
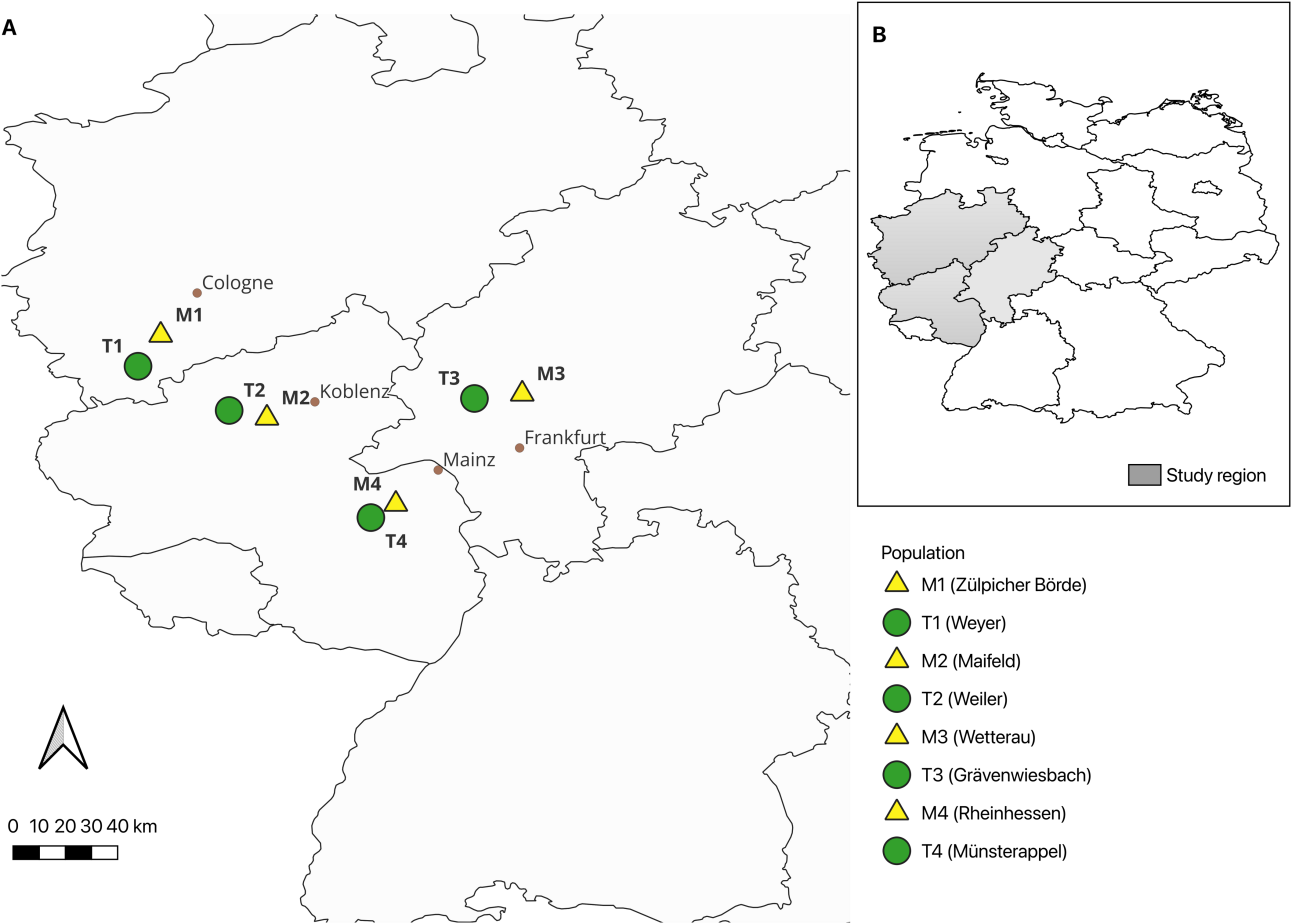
Location of the modern (yellow symbols) and traditional (green symbols) landscapes (A). The inset (B) shows the location of the study area within Germany (Rhineland‐Palatinate, Hesse and North Rhine‐Westphalia). The map was created using QGIS version 3.22 (QGIS Development Team 2023). Administrative boundaries are based on Natural Earth data (naturalearthdata.com).

Sampling was conducted in western Germany in four replicated landscape pairs, each consisting of a “modern” (M) and a “traditional” (T) agricultural landscape (Deppe et al. 2023, 2024, 2026) (Figure 2). The resulting eight landscapes were all ca. 11 × 9 km large. The minimum distance between the two landscapes within a pair ranged between 8.1 and 12.4 km (see Table A1). The minimum distance between the centers of two landscape pairs varied between 43 km (pairs 1 and 2) and 127 km (pairs 1 and 4). Modern and traditional landscapes differed in their composition and configuration, as detailed in Deppe et al. (2023, 2026). Compared to traditional landscapes, modern landscapes were characterized by higher proportions of crop fields (60%–81% vs. 36%–50%) and lower proportions of grassland (4%–9% vs. 13%–28%) and forests (1%–20% vs. 32%–51%). Regarding configuration, the mean size of crop fields was larger in modern (5.1 ha) than in traditional (2.7 ha) landscapes, while it was the other way round for grassland patches (modern: 0.8 ha, traditional: 1.5 ha). In the period 2013–2021, mean annual precipitation was 564 mm and mean temperature 11.1°C for pair 1, 544 mm and 11.3°C for pair 2, 572 mm and 11.2°C for pair 3, and 592 mm and 10.5°C for pair 4 (German Weather Service 2021).

Within each landscape, butterflies were collected at 10 sites, resulting in 80 sampling sites in total (for the respective coordinates see Deppe et al. 2023). Sampling sites within landscapes were, on average, 3.8 km apart from each other. We sampled between 18 and 22 individuals per site in the years 2021 and 2022. Field‐caught butterflies were transferred to the laboratory, kept for 2 days in a climate cabinet with a constant temperature of 22°C, and were eventually frozen at −80°C for later analyses. For genetics, we used eight butterflies per site, resulting in 640 individuals. For each sampling site, we assessed landscape composition and configuration within circular buffers with a radius of 250 m and 500 m (see Deppe et al. 2024 for details). In short, the proportional cover (%) of 15 land use categories (e.g., crop fields, grassland, forest) was measured using QGIS 3.22 (QGIS Development Team 2023). Compositional landscape heterogeneity (diversity of habitat types) was assessed as the Shannon diversity index of all 15 land cover types. The landscape configuration was characterized using patch‐based metrics, including mean patch size, patch distances, and perimeter to area ratios for crop fields and grassland.

### 2.3 DNA Extraction and ddRAD Library Preparation

Frozen butterflies were dissected, and the thoraces were used to isolate nuclear DNA with the DNeasy Blood & Tissue Kit (Qiagen, Germany) according to the manufacturer's instructions, but including additionally RNase (Qiagen, Germany). The DNA concentration and quality were measured using a Qubit 4 fluorometer, the dsDNA Broad Range Qubit Assay Kit (Thermo Fisher Scientific), and a 0.8% agarose gel. To determine the best combination of restriction enzymes and fragment size distribution, in silico digestion was performed on the *P. rapae* reference genome (GCF_905147795.1), derived from the NCBI database (https://www.ncbi.nlm.nih.gov/datasets/genome/?taxon=64459), using ddgRADer (Lajmi et al. 2023). Among several enzyme pairs evaluated, the pair EcoRI and NlaIII produced the highest number of fragments (43,752 with 370–600 bp), providing sufficient genomic representation for subsequent analyses. Consequently, this enzyme pair was used for double‐digest restriction site‐associated DNA sequencing (ddRADseq).

For genomic library preparation, we followed the protocol of Peterson et al. (2012). In brief, 120 ng of DNA was digested with 10 units of EcoRI‐HF and NlaIII with rCutSmart buffer (New England Biolabs). The digestion was incubated at 37°C for 3 h, and the products were cleaned with AMPure XP beads (Beckman Coulter) with 1.5× concentration. Afterwards, the reactions were ligated to a specific combination of P1 and P2 adapters (Peterson et al. 2012), using Hi‐T4 DNA ligase (New England Biolabs). The ligated reactions were pooled and cleaned with 1.5× AMPure XP beads. They were then subjected to 370–600 bp size selection on a 2% pre‐cast agarose gel on a PippinHT (Sage Science). The size‐selected samples were QC‐checked on a Bioanalyzer 2100 (HS dsDNA Assay, Agilent Technologies) and a Qubit 4 fluorometer (1X dsDNA HS Assay; Thermo Fischer Scientific) at the Institute of Molecular Biology, Mainz, Germany. Afterwards, we performed PCR enrichment to add Illumina flowcell annealing sequences using Phusion Plus DNA polymerase with eight cycles of amplification (ThermoFischer Scientific). We pooled the 640 individuals into 16 libraries (40 per library), using combinatorial barcoding (Peterson et al. 2012). The final libraries were cleaned using AMPure XP beads and quantified using a Qubit 4 fluorometer (1X dsDNA HS Assay; Thermo Fischer Scientific). The libraries were sequenced on an Illumina‐NovaSeq X Plus (Novogene GmbH, Germany), generating 150 bp paired‐end reads.

### 2.4 Assembly and SNP Calling

We used the Stacks 2.4 pipeline to assemble RAD loci and call single nucleotide polymorphisms (SNPs) from the raw ddRADseq data (Catchen et al. 2013). Raw reads were demultiplexed using the Process_Radtags program while discarding low‐quality reads (phred score > 10) and removing the restriction site tags. We obtained an average of 2.24 million reads per individual. After demultiplexing, read quality was checked using FastQC (Andrews et al. 2010) and summarized using MultiQC (Ewels et al. 2016). The paired‐end reads were assembled using the Burrows‐Wheeler Aligner (Li and Durbin 2009), using BWA‐MEM alignment with the reference genome of *P. rapae* . The alignments were converted to BAM files and sorted and indexed using SAMtools v1.18 (Li et al. 2009). We then used the Ref_Map program of Stacks. Individuals with > 25% missing data and coverages less than 20× were excluded from further analyses (*n* = 38; see Cerca et al. 2021). The mean effective coverage per sample of the remaining 602 individuals was 30×.

For the remaining individuals, the Population program within Stacks was once again run to retain SNPs present in ≥ 80% of the samples. Additional filtering options included (1) retaining only a single SNP per RADtag, (2) a minor allele frequency threshold of 0.05, (3) a maximum observed heterozygosity of 0.7, and (4) a minimum minor allele count of 3. Further filtering was done using VCFtools (Danecek et al. 2011). Variants with more than 10% missing genotype data and significant deviation (*p* < 0.001) from the Hardy–Weinberg equilibrium were excluded. The final dataset contained 11,381 SNPs. To assess whether pooled HWE filtering influenced the inference of population structure, we repeated the PCA and ADMIXTURE analyses without HWE filtering, while leaving the remaining pipeline unchanged. As this did not change any conclusion (Tables A2 and A3; Figure A3), only the results based on the analyses including HWE filtering are shown. SNPs were functionally annotated using SnpEFFv5.1d (Cingolani et al. 2012). The annotation was performed against the *P. rapae* reference genome version GCF_905147795.1, using the corresponding SNPeff genome database. The resulting annotated SNPs were parsed in R using the vcfR and Tidyverse packages to extract and summarize variant effects. Each SNP was categorized by its functional impact (high, moderate, low, modifier) and genomic region (coding, intronic, UTR, intergenic). Further, marker information index, observed heterozygosity, and minor allele frequency per locus were calculated using the Adegenet package in R (Jombart 2008). Chromosome‐level SNP densities were calculated by intersecting SNP positions with the official RefSeq assembly length data. SNP counts and densities (SNPs per megabase, Mb) were derived for each chromosome. Ti/Tv ratio was calculated based on substitution type and distribution of SNP effects. All bioinformatic work was carried out on a high‐performance computing server at the University of Kaiserslautern‐Landau (RPTU; https://hpc.rz.rptu.de/elwetritsch).

### 2.5 Analysis of Relatedness

To avoid biased population‐structure estimations, we performed linkage disequilibrium pruning with Plink v1.9.0‐b.7.11 to remove correlated SNPs (Purcell et al. 2007; Chang et al. 2015). We calculated pairwise relatedness among individuals using Coancestry (Wang 2011) and the Triadic Likelihood Estimator (TrioML). In TrioML, estimates of ≥ 0.250 indicate probable full‐siblings, 0.125–0.249 half‐siblings, and 0.060–0.124 cousins. Accordingly, we found 6 pairs of probable full‐siblings, of which one individual was excluded from further downstream analyses. Thus, the final data set comprised 596 individuals and 10,997 SNPs (see Dhyani et al. 2026).

### 2.6 Genetic Diversity

Genetic diversity within populations was assessed in R version 4.4.2 (R Core Team 2024), using the packages Adegenet (Jombart 2008) and Hierfstat (Goudet 2005). SNP data were used to estimate the number of loci, observed heterozygosity, expected heterozygosity, effective number of alleles, and inbreeding coefficient for each population. We further calculated private alleles and allelic richness within populations, the latter using a rarefaction approach to account for slight variation in sample size across populations.

### 2.7 Population Structure

AMOVA was used to quantify hierarchical genetic differentiation across multiple scales: among landscape pairs, landscapes within pairs, sampling sites within landscapes, individuals within sites, and within individuals. SNP data were processed in R using Adegenet (Jombart 2008), Poppr (Kamvar et al. 2014), and Ade4 (Dray and Dufour 2007). The genotypic data were converted to GenePop format using PGDSpider version 3.0.0.0 (Lischer and Excoffier 2012). To test for the significance of variance components and phi statistics, we used 999 permutations in the Ade4:randtest() function. To further assess genetic differentiation, pairwise *F*_ST_ values among populations were estimated using the StAMPP package (Pembleton et al. 2013) in R, with 1000 bootstrap replicates.

To explore the genetic population structure, a principal component analysis (PCA) was performed using Plink v1.9.0‐b.7.11 and its option to compute the first 100 principal components (Purcell et al. 2007; Chang et al. 2015). Eigenvalues and eigenvectors generated by Plink were used for downstream analysis and visualized by the Tidyverse package in R (Wickham et al. 2019). Individual ancestry proportions were estimated using Admixture for *K* = 1–10 ancestral populations (Alexander et al. 2009). For each *K*, five independent runs were performed to assess consistency across replicates. Model fit was evaluated using the admixture cross‐validation (CV) procedure, and CV errors were compared among *K* values to identify the optimal number of ancestral clusters. Ancestry proportion matrices (Q files) were used for visualization by the Tidyverse package in R (Wickham et al. 2019). To further assess genetic clustering and separation of populations, we conducted a discriminant analysis of principal components (DAPC) in R using Adegenet (Jombart 2008). Initial exploratory clustering was conducted with Find.clusters(), testing for up to 10 groups, to explore unsupervised structure, followed by supervised DAPC using known population labels. The optimal number of principal components was determined via cross‐validation (xvalDapc()), and seven discriminant functions were retained. The relationship between genetic and geographic distances was calculated using the Mantel test implemented in the Vegan package in R (Oksanen et al. 2022). The Mantel test was performed using both Pearson and Spearman correlation coefficients. Statistical significance was evaluated using 9999 permutations.

Finally, we tested for associations between environmental variables and genetic variation with redundancy analyses (RDAs) using the Vegan package in R (Oksanen et al. 2022). The landscape variables quantified for each sampling site were first standardized and then subjected to PCAs, separately for each scale (250 and 500 m buffers) to reduce dimensionality and minimize multicollinearity among variables. For each spatial scale, the first two principal components, explaining the majority of environmental variance, were retained as composite environmental variables representing landscape configuration and composition, respectively. We fitted standard and partial RDA models with genotype, PC1 and PC2 for each spatial scale, the latter additionally including the geographic coordinates as conditioning variables, thereby accounting for spatial autocorrelation. These models were fitted across all sampling sites simultaneously (i.e., all landscapes were combined without accounting for pairing). To directly test for genetic differentiation between landscape types, we performed an additional partial RDA contrasting modern and traditional landscapes. To account for the paired sampling design, the landscape pair was included as a conditioning factor. For all RDAs, significance was tested using 999 permutations. The proportion of genetic variance explained by environmental variables was quantified using both adjusted and unadjusted *r* ^2^ values.

## 3 Results

### 3.1 SNP Mining

The majority (82.5%) of the 11,381 SNPs were classified as having a modifier impact, typically associated with the intronic, intergenic, or upstream/downstream variants (see Figure A1). Only two SNPs were identified as high impact, involving stop‐gained and splice‐donor variants. The most frequent effect types were intronic variants (35.0%), upstream gene variants (21.3%), and synonymous variants (13.7%). SNP counts per chromosome ranged from 385 to 835, and densities from 36.9 to 69.5 SNPs per megabase (Mb; Figure A2). The transition/transversion ratio was 1.1, indicating a slight excess of transitions (5953) over transversions (5428). On average, the SNP markers showed moderate informativeness, with polymorphism information content (PIC) values ranging from 0.09–0.38 and minor allele frequencies (MAF) from 0.05 to 0.50. The mean observed heterozygosity (H_o_) across markers was 0.220.

### 3.2 Genetic Diversity

All diversity metrics were highly consistent across populations, including number of loci (10,509–10,719), observed heterozygosity (0.218–0.225), expected heterozygosity (0.229–0.236), effective number of alleles (1.298–1.309), and inbreeding coefficients (0.044–0.059; Table 1). Rarefied allelic richness was virtually identical across populations, ranging from 1.994 to 1.996 alleles per locus. No private alleles were detected in any of the populations.

**TABLE 1:** Summary of genetic diversity indices.

Population	*N*	N_loc_	H_o_	H_e_	N_e_	FIS	AR
Modern 1	79	10,509	0.218	0.229	1.298	0.049	1.994
Traditional 1	78	10,605	0.218	0.233	1.303	0.059	1.995
Modern 2	69	10,625	0.225	0.236	1.309	0.046	1.996
Traditional 2	77	10,674	0.222	0.236	1.309	0.058	1.996
Modern 3	73	10,533	0.220	0.231	1.300	0.044	1.994
Traditional 3	70	10,667	0.223	0.235	1.307	0.049	1.996
Modern 4	77	10,624	0.221	0.234	1.306	0.054	1.995
Traditional 4	73	10,719	0.224	0.236	1.309	0.050	1.996
Mean values		10,619	0.221	0.234	1.305	0.051	1.995

*Note:* Data is given for eight *Pieris rapae* populations based on ddRAD‐derived single nucleotide polymorphisms (SNPs).

Abbreviations: AR, allelic richness; FIS, inbreeding coefficient; H_e_, expected heterozygosity; H_o_, observed heterozygosity; N, number of individuals; N_e_, effective number of alleles; N_loc_, number of SNP loci.

### 3.3 Population Structure

AMOVA revealed that most genetic variation occurred within individuals (95.2%), followed by variation among individuals within sites (4.7%) and among sampling sites (0.04%). No significant variation was found among landscape pairs and among landscapes within pairs (Table 2). Accordingly, pairwise *F*_ST_ values were extremely low across all populations (0.00001–0.00038; Table 3), indicating negligible differentiation. Mantel tests showed no significant isolation by distance (Pearson: *r* = −0.018, *p* = 0.547; Spearman: *r* = −0.076, *p* = 0.654), indicating that geographic distance did not explain genetic differentiation in the tested populations.

**TABLE 2:** Analysis of molecular variance (AMOVA) for different sources of variation.

Source	DF	SSq	VarComp	Var %	Phi	*p*
Landscape pairs	3	5729	< 0.1	< 0.1	1.7 × 10^−6^	0.576
Landscapes [pairs]	4	7641	0.1	< 0.1	8.0 × 10^−5^	0.150
Sites [landscapes]	72	135,990	0.7	< 0.1	3.7 × 10^−4^	**0.022**
Individuals [sites]	516	969,527	85.1	4.7	0.047	**0.001**
Individuals	596	1,018,362	1708.7	95.2	0.048	**0.001**

*Note:* Results are based on SNPs for *Pieris rapae* from eight agricultural landscapes. Sources include variation among landscape pairs, landscapes within pairs, sampling sites within landscapes, indviduals within sampling sites, and within individuals. Significance was assessed using 999 permutations. Significant *p*‐values are given in bold.

Abbreviations: VarComp, variance component; Var %, variance in percent.

**TABLE 3:** Pairwise *F*_ST_ values between the investigated *Pieris rapae* populations.

	M1	T1	M2	T2	M3	T3	M4	T4
M1	—	< 0.001	< 0.001	0.001	0.015	0.007	0.009	< 0.001
T1	0.00029	—	< 0.001	0.005	< 0.001	< 0.001	0.016	0.011
M2	0.00031	0.00038	—	0.391	< 0.001	0.022	0.173	0.084
T2	0.00026	0.00018	0.00003	—	< 0.001	0.077	0.158	0.374
M3	0.00020	0.00032	0.00036	0.00033	—	< 0.001	0.002	0.001
T3	0.00020	0.00030	0.00017	0.00012	0.00028	—	0.279	0.214
M4	0.00018	0.00018	0.00008	0.00008	0.00029	0.00005	—	0.854
T4	0.00027	0.00019	0.00012	0.00003	0.00027	0.00007	0.00001	—

*Note:* The pairwise *F*_ST_ values are shown below the diagonal, and the corresponding *p*‐values above the diagonal.

Abbreviations: M, modern landscape; T, traditional landscape.

**FIGURE 3:**
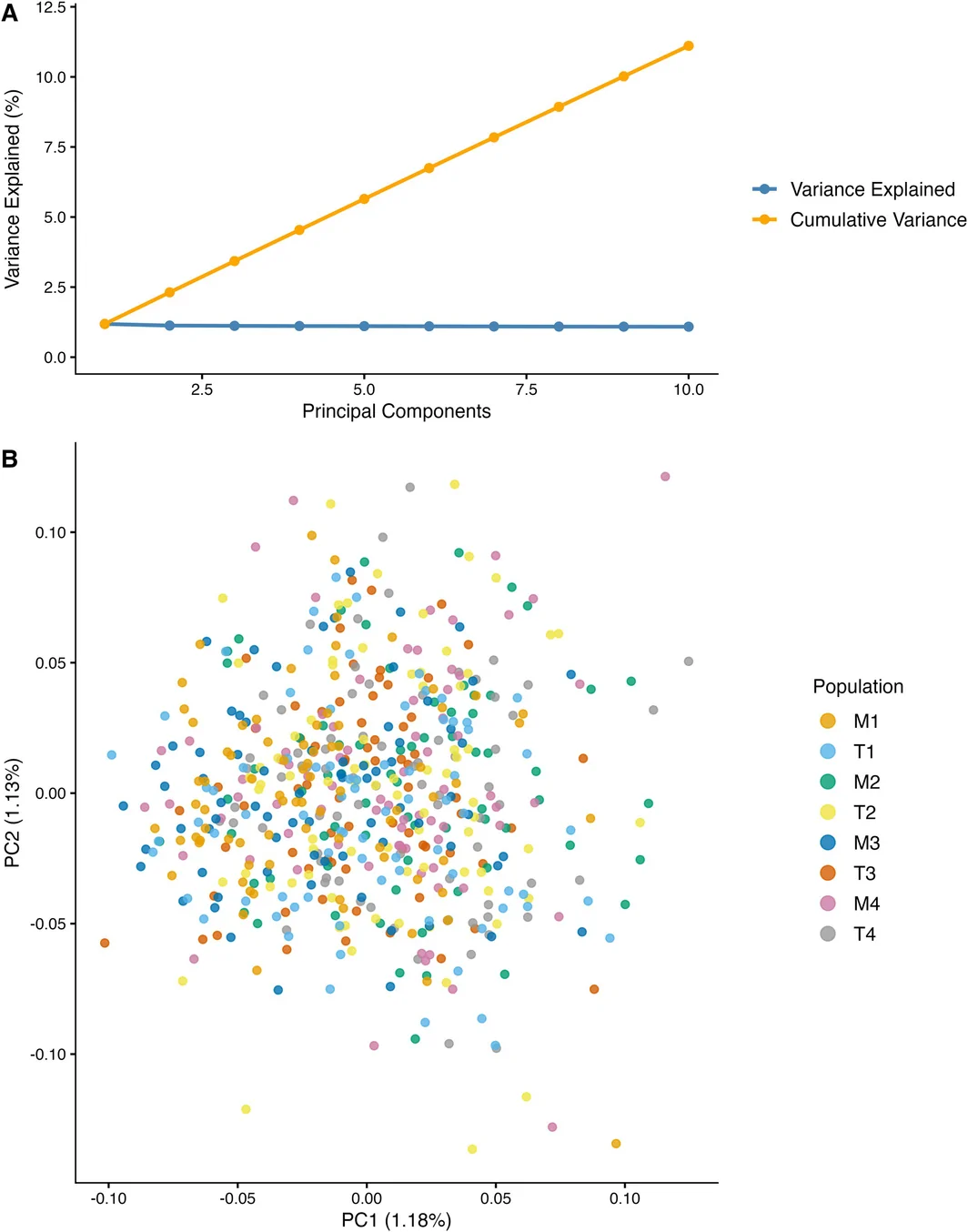
Principal component analysis (PCA) of genetic variation among eight *Pieris rapae* populations from modern (M) or traditional (T) landscapes. (A) Scree plot showing the percentage of genetic variation explained by the first 10 principal components. (B) Scatterplot of individuals against PC1 and PC2, colored by landscape.

**FIGURE 4:**
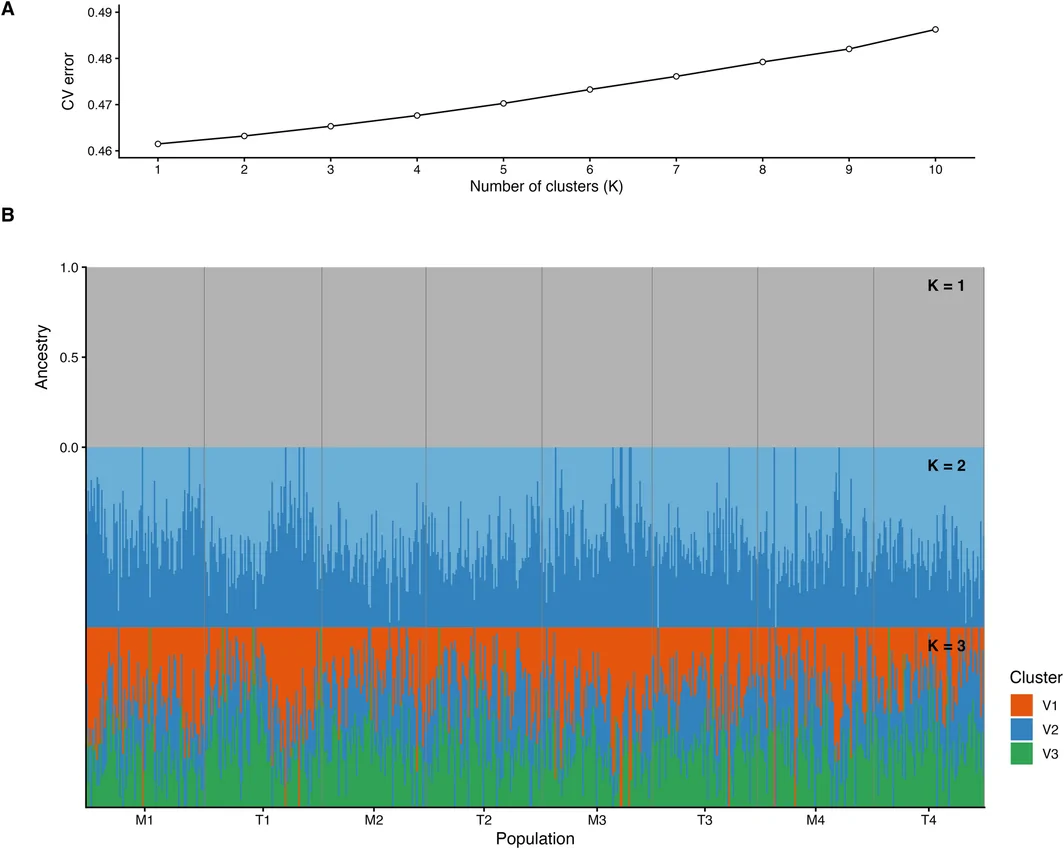
Population genetic structure for eight *Pieris rapae* populations, based on an admixture analysis of ddRAD‐derived single nucleotide polymorphisms. (A) Cross‐validation (CV) error across *K* = 1–10. (B) Individual ancestry proportions for *K* = 1–3. Each individual is represented by a vertical line, with colors indicating inferred ancestry components. Individuals are ordered by populations (M, modern landscape; T, traditional landscape). Although *K* = 2 and *K* = 3 are also shown, CVs supported *K* = 1, indicating the absence of a detectable population structure.

The PCA analysis showed that the top 10 principal components explained a cumulative variance of only 11.1%, indicating weak overall genetic differentiation among individuals and populations (Figure 3A). All populations showed substantial overlap with no evident clustering (Figure 3B). Nevertheless, weak latitudinal variation was detected. At the individual level, PC1 was negatively correlated with latitude (*r* = −0.196, *p* < 0.0001), and also at the population level, mean PC1 scores tended to decline northward (*β* = −0.025 ± 0.012, *r* ^2^ = 0.44, *p* = 0.075), which may suggest a weak north–south genetic gradient in allele frequencies. An admixture analysis identified *K* = 1 as the best supported model based on the lowest cross‐validation error, indicating no population structure (Figure 4).

**FIGURE 5:**
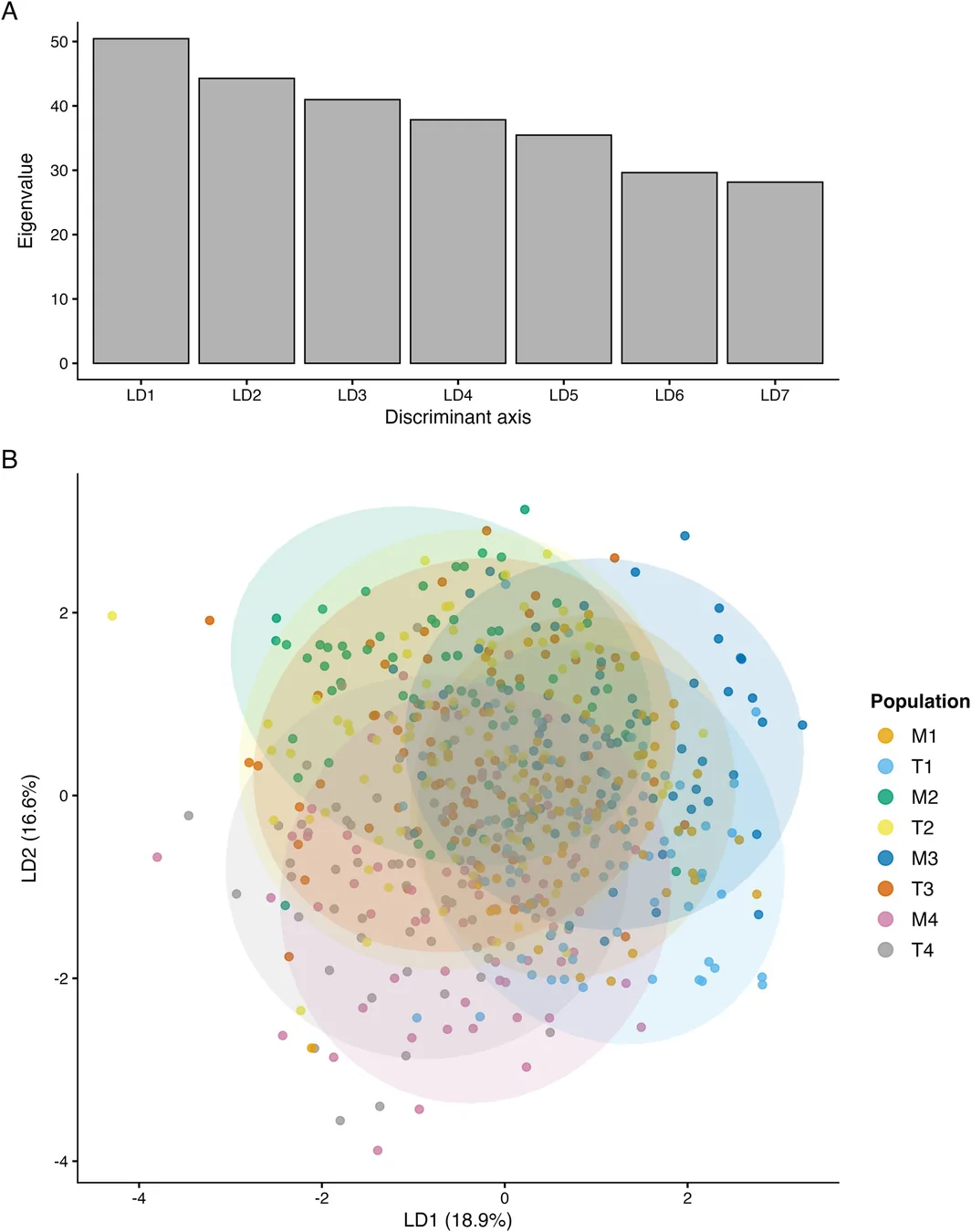
Discriminant analysis of principal components (DAPC) for genetic variation among eight *Pieris rapae* populations. (A) Eigenvalues of the retained discriminant functions, indicating the relative contribution of each linear discriminant (LD) to between‐group variation. (B) Scatterplot of individuals along the first two discriminant functions. Points represent individuals, and colors denote populations (M, modern landscape; T, traditional landscape). Ellipses indicate 95% confidence intervals for each population.

Redundancy analyses (RDAs) without accounting for the paired design revealed no significant genome‐wide associations between environmental variables and genetic variation, except for a weak but significant effect of landscape configuration (PC2) in the 500 m buffer model (Table 4). Likewise, the paired RDA comparing modern and traditional agricultural landscapes revealed only a weak effect of PC1. Overall, environmental variables explained less than 0.4% of the total genomic variation. Consequently, the discriminant analysis of principal components (DAPC) showed substantial overlap among populations with no clear separation along the first two discriminant functions (Figure 5). The eigenvalue distribution indicated a gradual decline across discriminant functions and no dominant axis of among‐group variation.

**TABLE 4:** Summary of redundancy analyses (RDAs).

Model	Model *p*	PC1 *p*	PC2 *p*	*r* ^2^	Adj. *r* ^2^
Regular RDAs					
250 m radius	0.149	0.494	0.062	0.0034	0.000043
500 m radius	0.116	0.542	**0.033**	0.0034	0.000049
Partial RDAs					
250 m radius	0.402	0.850	0.081	0.0034	0.000001
500 m radius	0.194	0.703	0.054	0.0034	0.000033
Partial RDA comparing landscape types					
Landscape type	0.055	**0.040**	—	0.0017	< 0.000001

*Note:* The RDAs tested for associations between landscape variables (as measured in a 250 and 500 m radius around the sampling sites) and genome‐wide genetic variation in *Pieris rapae* populations. The partial (spatial) models include latitude and longitude as conditioning variables, while the regular models do not. The final row represents a paired RDA, contrasting modern and traditional landscapes while conditioning on replicate pairs and therefore including only a single constrained axis. All other RDAs were run across all sampling sites. PC1 represents landscape composition and agricultural intensification, and PC2 landscape configuration and fragmentation. *r* ^2^ and adjusted *r* ^2^ represent the proportion of total genetic variance explained by environmental variables. Significant *p*‐values are given in bold.

## 4 Discussion

Our genome‐wide SNP analysis revealed a lack of population genetic structure in *P. rapae* across western Germany. Genetic differentiation among populations was negligible, as indicated by extremely low pairwise *F*_ST_ values, absence of isolation by distance, and support for a single genetic cluster. Moreover, genetic diversity metrics were highly similar across populations, with no private alleles detected and virtually identical allelic richness. Thus, we essentially found a panmictic population structure at the spatial scale considered.

These patterns are consistent with theoretical expectations for species characterized by high dispersal ability and ecological generalism. In such species, gene flow can counteract genetic drift, homogenizing allele frequencies across space and preventing the emergence of spatial genetic structure (Bohonak 1999; Lenormand 2002; Lowe and Allendorf 2010). The absence of isolation by distance further supports this interpretation, as genetic similarity would be expected to decline with geographic distance under restricted dispersal (Wright 1943; Slatkin 1993). In our study, the lack of such a pattern suggests effective dispersal across the entire study region. Our findings are also in line with earlier results on the same species. Ryan et al. (2019) reported the absence of detectable genetic structure in European *P. rapae* populations. Deppe et al. (2023) used the same four landscape pairs, revealing no effects of fragmentation on condition and dispersal‐related traits in *P. rapae* . Thus, the absence of detectable variation in both genomic and phenotypic data strongly supports the notion of high dispersal ability resulting in a panmictic population.

Importantly, our paired design allowed us to test whether contrasting agricultural landscape structures influence genetic connectivity. We found no evidence that highly intensified, modern landscapes exhibit stronger genetic structuring than more traditional, heterogeneous landscapes, thereby rejecting one of our a priori hypotheses. Our analyses revealed no significant differentiation among landscape types or pairs, indicating that pronounced differences in habitat configuration do not translate into detectable genetic structuring in *P. rapae* (cf. Fahrig 2003; Jackson and Fahrig 2015).

Redundancy analyses further indicated that contemporary landscape composition and configuration had at best minimal effects on genomic variation in *P. rapae* . Environmental variables explained less than 0.4% of total genomic variance; associations between genetic variation and landscape structure were very weak. This pattern remained unchanged when accounting for the paired design, reinforcing the conclusion that landscape‐level differences in fragmentation had negligible effects on genome‐wide variation. In line with landscape genetic theory, the strength of such effects is expected to depend strongly on species‐specific traits, particularly dispersal ability (Manel et al. 2003; Ewers and Didham 2006; Storfer et al. 2010).

Our findings contrast with several studies demonstrating strong genetic structuring in less mobile or more specialized butterfly species inhabiting fragmented landscapes. In such species, limited dispersal and strong habitat specificity often result in reduced gene flow, increased genetic differentiation, and elevated extinction risk (Hanski 1999; Keyghobadi 2007; Krauss et al. 2003). In contrast, generalist species associated with human‐modified environments frequently maintain high connectivity and may persist or even expand in agricultural landscapes (Dennis et al. 2003; Clavel et al. 2011). Our results for *P. rapae* are consistent with this broader pattern of trait‐mediated responses to environmental change, highlighting the role of ecological generalism in buffering against landscape modification.

Despite the overall lack of structure, we detected a weak latitudinal gradient in genetic variation, as indicated by the correlation between PC1 and latitude at the individual level. Although this pattern was subtle, it may reflect large‐scale processes such as spatially varying selection or historical colonization dynamics (Endler 1977; Slatkin 1993). However, given the low overall differentiation and the rather small geographic distances, this gradient should be interpreted cautiously. On principle, it could indicate some weak adaptive variation along broader climatic gradients not captured by the environmental variables considered here.

Overall, our results demonstrate that extensive anthropogenic habitat fragmentation does not necessarily translate into reduced genetic connectivity in highly mobile, generalist species. In *P. rapae* , high dispersal ability and ecological flexibility appear sufficient to maintain panmictic population structure across a heterogeneous, human‐dominated landscape. Even under a replicated paired design explicitly contrasting different agricultural landscape types, no evidence for reduced connectivity in more fragmented systems was detected. These findings highlight the importance of species‐specific life‐history traits in shaping genetic responses to global environmental change.

## Author Contributions

**Praveen Dhyani:** conceptualization (supporting), data curation (lead), formal analysis (lead), investigation (equal), methodology (equal), validation (lead), visualization (lead), writing – review and editing (equal). **Franziska Deppe:** data curation (supporting), investigation (equal), writing – review and editing (equal). **Klaus Fischer:** conceptualization (lead), funding acquisition (lead), investigation (supporting), methodology (equal), project administration (lead), resources (lead), supervision (lead), writing – original draft (lead).

## Funding

This work was supported by the Deutsche Forschungsgemeinschaft (DFG, German Research Foundation)—FI 846/14‐1.

## Conflicts of Interest

The authors declare no conflicts of interest.

## Supporting information


**Table A1:** Coordinates for sampling sites of *Pieris rapae* populations in western Germany.
**Table A2:** Sensitivity of population structure inference to pooled Hardy–Weinberg equilibrium (HWE) filtering.
**Table A3:** HWE deviations within landscapes compared with the pooled dataset.
**Figure A1:** Functional annotation and substitution profile of ddRAD‐derived single‐nucleotide polymorphisms (SNPs) identified in *Pieris rapae* . (A) Distribution of SNPs across impact categories. (B) Distribution of SNP base substitution types, the inset pie chart summarizes the transitions vs. traversions ratio. (C) Functional impact categories of SNPs. (D) Top 10 annotated SNP effect classes based on functional annotation.
**Figure A2:** Chromosomal distribution of ddRAD‐derived single‐nucleotide polymorphisms (SNPs) across the *Pieris rapae* genome. (A) Heatmap showing the distribution of SNPs using a non‐overlapping 1 megabase (Mb) window. (B) Number of SNPs detected per chromosome. (C) Relationship between chromosome length (Mb) and SNP count.
**Figure A3:** Principal component analysis (PCA) of genetic variation among eight *Pieris rapae* populations after omitting the pooled Hardy–Weinberg equilibrium (HWE) filtering. The analysis was otherwise performed using the same sample exclusions and LD‐pruning procedure as in the original analysis, resulting in 20,208 SNPs. Panel (A) shows the variance explained by the first 10 principal components and cumulative variance. Panel (B) shows individual scores on PC1 and PC2, colored by landscape. PC1 and PC2 explained 1.182% and 1.096% of the total genetic variation, respectively, and populations showed substantial overlap.

## Data Availability

Raw ddRAD sequencing reads for all 640 sampled *Pieris rapae* individuals have been deposited in the NCBI Sequence Read Archive under BioProject accession PRJNA1462033. The final filtered SNP dataset used for downstream analyses (596 individuals; 10,997 SNPs), associated sample metadata, and documentation of excluded individuals have been deposited in the Dryad Digital Repository: https://doi.org/10.5061/dryad.g79cnp65w.
