# Supplementary material for: Panmixia in a Widespread Butterfly: High Dispersal and Ecological Generalism Buffer Against Landscape Fragmentation
Source: Ecol Evol. 2026 Sep 3;16(9):e74318. doi: 10.1002/ece3.74318 (PMC13542571; doi:10.1002/ece3.74318)
Supplement: Supplementary file 1 [file ECE3-16-e74318-s001.docx]

**Appendix**

**Table A1:** Coordinates for sampling sites of *Pieris rapae* populations in western Germany.

| **Replicate pair** | **Modern landscape (M)** | **Traditional landscapes (T)** | **Federal state** | **Region** | **Distance (km)** |
| --- | --- | --- | --- | --- | --- |
| 1 | Zülpicher Börde  (50°42′45.9″ N, 6°45′56.3″ E) | Weyer  (50°32′48.1″ N, 6°38′40.3″ E) | NRW /  Rhineland-Palatinate | Western Germany | 12.4 |
| 2 | Maifeld  (50°16′13.7″ N, 7°19′58.1″ E) | Weiler  (50°18′38.9″ N, 7°07′52.3″ E) | Rhineland-Palatinate | Southwest Germany | 9.8 |
| 3 | Wetterau  (50°23′55.5″ N, 8°41′46.1″ E) | Grävenwiesbach  (50°22′29.2″ N, 8°26′27.3″ E) | Hesse | West-central Germany | 9.8 |
| 4 | Rheinhessen  (49°48′39.6″ N, 8°01′16.2″ E) | Münsterappel  (49°44′21.5″ N, 7°53′16.3″ E) | Rhineland-Palatinate | Southwest Germany | 8.1 |

The table includes federal state, region and geographic distances between modern and traditional landscapes within landscape pairs. NRW: North Rhine-Westphalia.

**Table A2:** Sensitivity of population structure inference to pooled Hardy-Weinberg equilibrium (HWE) filtering.

| **Metric** | **HWE-filtered analysis** | **No HWE filtering** | **Interpretation** |
| --- | --- | --- | --- |
| SNPs after LD prunning | 10,997 | 20,208 | Nearly doubled marker set |
| PC1 variance (%) | 1.185 | 1.182 | Essentially unchanged |
| PC2 variance (%) | 1.127 | 1.096 | Essentially unchanged |
| First 10 PCs cumulative (%) | 11.107 | 10.902 | Essentially unchanged |
| ADMIXTURE best-supported K | 1 | 1 | Same conclusion |
| ADMIXTURE CV error at K = 1 | 0.461 | 0.512 | K = 1 had the lowest CV error in both analyses |

Population structure analyses were repeated without the pooled HWE filter using the same downstram sample exclusions and LD-prunning settings as in the original analysis. This did not change any conclusion.

**Table A3:** HWE deviations within landscapes compared with the pooled dataset.

| **Landscape** | **SNPs tested** | **HWE failures** | **Failure (%)** | **Het. Deficit** | **Het. excess** |
| --- | --- | --- | --- | --- | --- |
| Modern 1 | 21,006 | 1,601 | 7.6 | 1,600 | 1 |
| Traditional 1 | 21,006 | 1,675 | 8.0 | 1,675 | 0 |
| Modern 2 | 21,006 | 1,266 | 6.0 | 1,266 | 0 |
| Traditional 2 | 21,006 | 1,669 | 8.0 | 1,669 | 0 |
| Modern 3 | 21,006 | 1,574 | 7.5 | 1,573 | 1 |
| Traditional 3 | 21,006 | 1,554 | 7.4 | 1,553 | 1 |
| Modern 4 | 21,006 | 1,674 | 8.0 | 1,674 | 0 |
| Traditional 4 | 21,006 | 1,472 | 7.0 | 1,471 | 1 |
| Pooled | 21,006 | 9,625 | 45.8 | 9,624 | 1 |

Among the 9,625 SNPs failing pooled HWE, 4,180 (43.4%) did not fail HWE in any individual landscape.

**Figure A1:** Functional annotation and substitution profile of ddRAD-derived single-nucleotide polymorphisms (SNPs) identified in *Pieris rapae*. A: Distribution of SNPs across impact categories. B: Distribution of SNP base substitution types, the inset pie chart summarises the transitions vs. traversions ratio. C: Functional impact categories of SNPs. D: Top 10 annotated SNP effect classes based on functional annotation.

**Figure A2:** Chromosomal distribution of ddRAD-derived single-nucleotide polymorphisms (SNPs) across the *Pieris rapae* genome. A: Heatmap showing the distribution of SNPs using a non-overlapping 1 megabase (Mb) window. B: Number of SNPs detected per chromosome. C: Relationship between chromosome length (Mb) and SNP count.

**Figure A3:** Principal component analysis (PCA) of genetic variation among eight *Pieris rapae* populations after omitting the pooled Hardy-Weinberg equilibrium (HWE) filtering. The analysis was otherwise performed using the same sample exclusions and LD-pruning procedure as in the original analysis, resulting in 20,208 SNPs. Panel A shows the variance explained by the first 10 principal components and cumulative variance. Panel B shows individual scores on PC1 and PC2, coloured by landscape. PC1 and PC2 explained 1.182% and 1.096% of the total genetic variation, respectively, and populations showed substantial overlap.
